# *Bulletin* comment: Openness, transparency and candour^†^

**DOI:** 10.1192/pb.bp.114.048546

**Published:** 2014-08

Health professionals are well known for being the worst patients. They refuse to seek help for difficulties that, if addressed earlier, might not have resulted in catastrophic complications for themselves and those around them including friends, family and indeed patients. With particularly high rates of psychiatric illness in those working in mental health, it is vital the reasons why those affected delay getting treatment are identified and eliminated.

Research exploring stigmatising beliefs and attitudes among colleagues in mental health services may help. In addition, the general public’s understanding of psychological issues will not change unless everyone - especially mental health professionals - has the courage to stand up and be counted when they are affected in the same way. Policy makers, agenda setters and strategy shapers should be aware of high levels of psychiatric illness among practitioners and any research confirming such facts ought to be used as a burning torch highlighting ways to combat it.

Personal difficulty, with or without diagnosable episodes of mental illness, is part of the human experience and needs to be acknowledged by all, but particularly by mental health professionals and organisations involved in the care of patients. Recognition of one’s own shortcomings paradoxically might lead to greater respect from both patients and society in general.

